# GMASS: a novel measure for genome assembly structural similarity

**DOI:** 10.1186/s12859-019-2710-z

**Published:** 2019-03-18

**Authors:** Daehong Kwon, Jongin Lee, Jaebum Kim

**Affiliations:** Department of Biomedical Science and Engineering, Konkuk University, Seoul, 05029 South Korea

**Keywords:** Measure, Genome, Assembly, Structural similarity

## Abstract

**Background:**

Thanks to the recent advancements in next-generation sequencing (NGS) technologies, large amount of genomic data, which are short DNA sequences known as reads, has been accumulating. Diverse assemblers have been developed to generate high quality de novo assemblies using the NGS reads, but their output is very different because of algorithmic differences. However, there are not properly structured measures to show the similarity or difference in assemblies.

**Results:**

We developed a new measure, called the GMASS score, for comparing two genome assemblies in terms of their structure. The GMASS score was developed based on the distribution pattern of the number and coverage of similar regions between a pair of assemblies. The new measure was able to show structural similarity between assemblies when evaluated by simulated assembly datasets. The application of the GMASS score to compare assemblies in recently published benchmark datasets showed the divergent performance of current assemblers as well as its ability to compare assemblies.

**Conclusion:**

The GMASS score is a novel measure for representing structural similarity between two assemblies. It will contribute to the understanding of assembly output and developing de novo assemblers.

**Electronic supplementary material:**

The online version of this article (10.1186/s12859-019-2710-z) contains supplementary material, which is available to authorized users.

## Background

Recent advances in next-generation sequencing (NGS) technologies have facilitated a rapid production of large amounts of short sequencing data from genomes, known as reads, at a low cost [1–3]. The length of reads is up to several hundred base pairs in the case of short read sequencing technologies such as Illumina, and up to several hundred kilo base pairs in the case of long read sequencing technologies such as Oxford Nanopore. Since the NGS reads are still much shorter than chromosomes lengthwise, a de novo assembly process is necessary to reconstruct the whole chromosome sequences [4]. The de novo assembly begins with finding and connecting overlapping NGS reads, which leads to the generation of longer sequences, known as contigs. Contigs are then ordered and oriented to construct much longer sequences, known as scaffolds, using paired-end or mate-pair reads generated from long DNA fragments.

Several algorithms have been developed to generate high-quality de novo assemblies. They are mainly classified into three categories: the greedy graph based algorithm, the Overlap-Layout-Consensus based algorithm, and the *de Bruijn* graph based algorithm [4]. The greedy graph based algorithm, such as SSAKE [5], VCAKE [6] and SHARCGS [7], incrementally connects a read to another read or contig with the highest overlap score until there is no more overlapping. The overlap scores are calculated using the number of matching bases in the overlap. Both the Overlap-Layout-Consensus based algorithm and *de Brujin* graph-based algorithm rely on a graph structure constructed from the NGS reads. They first construct an overlap graph using overlap information obtained from the NGS reads, and then the de novo assembly is constructed by the traversing the graph. However, the Overlap-Layout-Consensus based algorithm, such as CABOG [8], Newbler [9] and Celera assemblers [10], constructs an overlap graph using the direct overlap among the NGS reads, whereas the *de Bruijn* graph based algorithm, such as ABySS [11], SOAPdenovo [12], ALLPATHS-LG [13] and Velvet [14], is based on the overlap of all possible subsequences of length k, known as k-mer, extracted from the NGS reads.

The performance of assemblers is quite different due to algorithmic differences, which results in difficulty in choosing the most appropriate assembler for different NGS data [15–17]. Many benchmarking studies, such as Genome Assembly Gold-standard Evaluations (GAGE) [18], the GAGE for bacterial organisms (GAGE-B) [19], and Assemblathon 1 and 2 [20, 21], have been performed using synthetic and real NGS read datasets with attempts to overcome this problem. In these studies, N50 is the most widely used measure to assess the level of assembly contiguity. N50 is a length-weighted median of ordered scaffolds based on their lengths. Here, the weight of a scaffold is defined as the length of the scaffold divided by the total length of an assembly. NG50 was introduced in the Assemblathon project to consider the genome size. NG50 is similar to N50 except for the fact that in NG50, the weight of a scaffold is not calculated using the length of an assembly but the known or estimated length of a genome. Also, for more comprehensive evaluation of assemblies, Feature-Response Curve was introduced and compared with the standard metrics, such as N50, coverage and contig sizes [22]. The accuracy of an assembly was measured by determining genomic dissimilarity by comparing it to the true assembly and by checking the presence of core eukaryotic genes [23] or single-copy orthologs [24] in the assembly. The mapping pattern of reads was also used for detecting mis-assemblies and measuring accuracy of assembly [25]. By comparing assemblies with these measures, several benchmarking studies have identified that the performance of assemblers varies considerably [18–22].

Even though these measures are good for evaluating the quality of a single assembly, they do not directly compare two different assemblies. For example, when two assemblies are compared using N50, researchers can identify how dissimilar they are in terms of the level of assembly fragmentation but cannot measure the fraction of highly similar assembly regions between them. This kind of comparison is especially useful when a de novo assembly of a certain species needs to be compared to a reference species, or when multiple assemblies of the same species generated by different assembly programs need to be compared to check how similar or different they are. Comparison of assemblies typically begins with their alignment. If there are large-scale assembly rearrangements, a large number of alignment fragments are normally obtained. Using alignment results, several measures including the number of alignment fragments, and alignment coverage have been separately used to measure assembly similarity. In general, the more similar two assemblies are, it is expected that the smaller number of alignment fragments are constructed between them and alignment coverage of assemblies are higher. However, the number of alignment fragments can also be reduced by generating low coverage alignments, and the alignment coverage can also be increased by generating large number of small alignment fragments. Therefore, a single quantitative measure combining those two is necessary. In addition, the alignment fragments from assemblies are generated based on a resolution parameter, which determines the minimum length of the alignment, and the choice of the resolution parameter is arbitrary.

In this perspective, a new measure, called GMASS, was developed for comparing the structures of two genome assemblies. The GMASS score was defined based on the distribution pattern of the number and coverage of similar regions between a pair of assemblies. Specifically, the alignments of two assemblies are first generated, and then the number of alignment fragments and their coverage are obtained. This process is repeated using a certain range of resolutions, and those two values from different resolutions are combined to generate a single quantitative score. The GMASS score was related to the structural similarity between assemblies when it was evaluated using simulated assembly datasets. The GMASS score was also applied to compare pairs of assemblies obtained from previous benchmarking studies [18, 20], and the results showed that the current assemblers generate very different assemblies even though the same sequencing read datasets are used.

## Methods

### The GMASS score

**Fig. 1:**
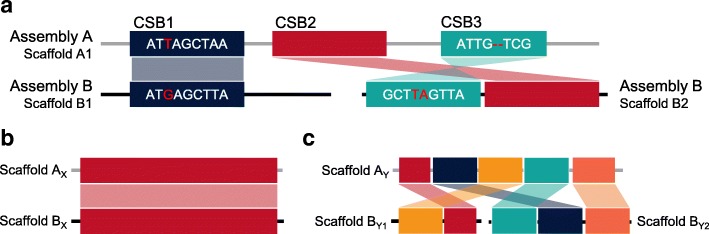
Examples of consensus segment blocks (CSBs). **a** CSBs are defined as aligned assembly regions between two assemblies. Sequences in CSBs can contain substitutions (red bases in CSB1) and indels (insertions and deletions; red parts in CSB3). The size of gaps between two CSBs is smaller than a chosen resolution (the minimum length of a CSB). **b** When two scaffolds from different assemblies are highly similar, a CSB constructed from them covers scaffolds almost entirely. **c** If the scaffolds from different assemblies are highly rearranged against to each other, a large number of CSBs with short length are constructed. In this case, their total size is almost equal to the total size of the scaffolds

The GMASS score represents the structural similarity of a pair of genome assemblies based on the length and number of similar genomic regions defined as consensus segment blocks (CSBs) in the assemblies. The CSBs are aligned assembly regions between two assemblies which can contain small number of substitutions and small gaps shorter than a chosen resolution (Fig. 1a). The way of constructing CSBs is described in the following subsection. The GMASS score is defined as Eq. 1 based on the assumption that the number and length of CSBs between two assemblies is equivalent to the number and length of sequence fragments (scaffolds or chromosomes) of the two assemblies as the two assemblies become more similar.

1 $$ GMASS=\frac{\ {\sum}_{i=r}^R{S}_i\ }{n}=\frac{\ {\sum}_{i=r}^R\left\{{L}_i\left({A}_1,{A}_2\right)\times {C}_i\left({A}_1,{A}_2\right)\right\}\ }{n} $$

The GMASS score is the average of structural similarity scores (*S*_*i*_) between two assemblies *A*_1_ and *A*_2_ across a certain range of CSB resolutions. In Eq. 1, *S*_*i*_ denotes the structural similarity score at the CSB resolution *i* belonging to a set of *n* different resolutions with a minimum value *r* and a maximum value *R*. *S*_*i*_ is calculated by the product of two terms related to the total length of the CSBs (*L*_*i*_(*A*_1_, *A*_2_)) and the number of CSBs (*C*_*i*_(*A*_1_, *A*_2_)).

*L*_*i*_(*A*_1_, *A*_2_) is the fraction of the total length of the CSBs against the total length of assemblies used to construct the CSBs with the CSB resolution *i* defined as Eq. 2. In Eq. 2, *l*(*A*_*x*_) is the total length of sequence fragments larger than resolution *i* in the assembly *A*_*x*_, and *l*(*CSBs*_*x*_) is the total length of blocks in the CSBs belonging to *A*_*x*_ defined as *CSBs*_*x*_.

2 $$ {L}_i\left({A}_1,{A}_2\right)=\frac{l\left({CSBs}_1\left)+l\right({CSBs}_2\right)}{l\left({A}_1\right)+l\left({A}_2\right)} $$

For example, suppose CSBs are constructed for the two scaffold assemblies *A*_1_ and *A*_2_ using 10 Kbp resolution. If total 2 Mbp and 2.5 Mbp scaffold sequences of *A*_1_ and *A*_2_ respectively are used in this construction, and total 1.8 Mbp and 2.2 Mbp scaffold sequences of *A*_1_ and *A*_2_ respectively are actually included in the CSBs, then *l*(*A*_1_)= 2 Mbp, *l*(*A*_2_)= 2.5 Mbp, *l*(*CSBs*_1_)= 1.8 Mbp, and *l*(*CSBs*_2_)= 2.2 Mbp. Therefore, *L*_10*Kbp*_(*A*_1_, *A*_2_) = (1.8 + 2.2)/(2 + 2.5) = 0.89.

*C*_*i*_(*A*_1_, *A*_2_) denotes the similarity between two assemblies in terms of the total number of CSBs constructed with the CSB resolution *i* defined as Eq. 3.

3 $$ {C}_i\left({A}_1,{A}_2\right)=\left\{{}_{1-\frac{c\left({CSB}_{S_1}\right)+c\kern0.22em \left({CSB}_{S_2}\right)-c\;\left({A}_1\right)-c\;\left({A}_2\right)}{\mathit{\max}c\;\left({A}_1\right)+\mathit{\max}c\;\left({A}_2\right)-c\;\left({A}_1\right)-c\;\left({A}_2\right)},\kern5.879990em otherwise}^{\frac{c\left({CSB}_{S_1}\right)+c\kern0.22em \left({CSB}_{S_2}\right)}{c\;\left({A}_1\right)+c\;\left({A}_2\right)},\kern1.2em if\;c\;\left({A}_1\right)+c\;\left({A}_2\right)\kern0.36em \ge c\kern0.24em \left({CSB}_{S_1}\right)+c\;\left({CSB}_{S_2}\right)}\kern0.36em \right. $$

Here *c*(*X*) is the number of sequence fragments larger than given resolution *i* (if *X* is an assembly) or the number of blocks constructed using the CSB resolution *i* (if *X* are CSBs). We note that if *X* are CSBs, then *c*(*CSBs*_1_) and *c*(*CSBs*_2_) are the same because of the definition of a CSB. *maxc*(*X*) is the maximum number of CSBs that can be constructed from an assembly *X* using the CSB resolution *i*. If the sum of the number of CSBs from two assemblies is less than or equal to the sum of sequence fragments larger than resolution *i* in the two assemblies, *C*_*i*_(*A*_1_, *A*_2_) is defined as the fraction obtained from those two numbers (the upper part of Eq. 3). Otherwise, the positive difference between those two numbers is calculated, and its fraction against a similar difference using the sum of the maximum number of CSBs that can be constructed using the two assemblies is obtained. The result is subtracted from 1 to make a positive correlation between *C*_*i*_(*A*_1_, *A*_2_) and assembly similarity (the lower part of Eq. 3). The maximum number of CSBs *maxc*(*A*_*x*_) of an assembly *A*_*x*_ is calculated by Eq. 4.

4 $$ maxc\left({A}_x\right)=\frac{l\left({A}_x\right)}{i} $$

Here *i* is the resolution used to construct CSBs. Eq. 4 implies that the minimum length of a CSB is *i*, and therefore at most *l*(*A*_*x*_)/*i* CSBs can be constructed. This was empirically validated using the GAGE and Assemblathon 1 dataset.

For example, suppose again that CSBs are constructed for the two scaffold assemblies *A*_1_ and *A*_2_ with *l*(*A*_1_)= 2 Mbp and *l*(*A*_2_)= 2.5 Mbp using 10 Kbp resolution. If total 20 and 24 scaffolds of *A*_1_ and *A*_2_ respectively are used in this construction, and total 20 CSBs are constructed, then *c*(*A*_1_)= 20, *c*(*A*_2_)= 24, and $c\left({CSB}_{S_1}\right)=$ $c\left({CSB}_{S_2}\right)$ = 20. In this case, *c*(*A*_1_) + *c*(*A*_2_) ≥ *c*(*CSBs*_1_) + *c*(*CSBs*_2_) holds, and therefore, *C*_10*Kbp*_(*A*_1_, *A*_2_)= (20 + 20)/(20 + 24) = 0.91 by the upper part of Eq. 3. On the other hand, if $c\left({CSB}_{S_1}\right)=$ $c\left({CSB}_{S_2}\right)$ = 25, the upper part of Eq. 3 cannot be used because *C*_10*Kbp*_(*A*_1_, *A*_2_)= (25 + 25)/(20 + 24) > 1 based on the upper part of Eq. 3 and the *C*_*i*_(*A*_1_, *A*_2_) score between 0 and 1 is needed. In this case, *C*_*i*_(*A*_1_, *A*_2_) is calculated by the lower part of Eq. 3 using *c*(*A*_1_)= 20, *c*(*A*_2_)= 24, $c\left({CSB}_{S_1}\right)=$ $c\left({CSB}_{S_2}\right)$ = 25, *maxc*(*A*_1_)= 2000/10 = 200, and *maxc*(*A*_2_)= 2500/10 = 250. Therefore, *C*_10*Kbp*_(*A*_1_, *A*_2_)= 1 – (25 + 25–20 – 24)/(200 + 250–20 – 24) = 0.99.

### Calculation of the GMASS score for simulated and benchmark datasets

In this study, CSBs were constructed from three datasets: simulated genome assemblies, GAGE [18], and Assemblathon 1 [20]. In the case of the simulated genome assemblies, the pairwise alignments of simulated genome assemblies (reference against 11 other assemblies with different divergence to the reference) were obtained from previous study [26]. This dataset simulated the evolution of two human chromosomes 21 and 22 from a common ancestor of 12 species with varying divergences. The synteny block generation program in inferCars [27] was then used to construct CSBs between each pair of assemblies with a given resolution.

In the case of the other two datasets, assembly sequences as a form of scaffolds were downloaded from a website with their datasets. Then repeats in the assemblies were first masked using RepeatMasker (version 4.0.5) [28], and pairwise alignments between two different assemblies were generated using LASTZ (version 1.02) [29] with parameters “-E=150 -H=2000 -K=4300 -L=2200 -M=254 -O=600 -T=2 -Y=15000”. In evaluation of the GMASS score, self-alignments of each assembly were also constructed using LASTZ parameters “-O=400 -E=30 -X=910 -Y=9400 -K=3000 -L=3000 -H=2000 -T=1 -Z=1”. These parameter values were based on the recommendation found in the UCSC genome browser [30]. Once pairwise alignments of genome assemblies were prepared, the synteny block generation program in inferCars [27] was used to construct CSBs between a pair of assemblies with a given resolution.

In the process of the CSB generation between two assemblies, one of the assemblies needs to be a reference. In the case of the simulated assembly dataset, a reference assembly specified in that study was also used as the reference in this study. Therefore, the CSBs of total 11 pairs (reference against 11 other assemblies) were constructed. In the GAGE and Assemblathon 1 dataset, a total of eight assemblies by eight assembly programs (ABySS2 [31], ALLPATHS-LG [13], Bambus2 [32], CABOG [8], SGA [33], SOAPdenovo [12], MSR-CA [34] and Velvet [14]) and five assemblies by five assembly programs (ABySS [11], ALLPATHS-LG [13], Celera [10], Meraculous [35] and SOAPdenovo [12]) exist respectively, and CSBs of all possible assembly pairs were constructed by using one assembly with a higher N50 as the reference. In terms of the resolution, five different resolutions (100, 200, 300, 400, and 500 Kbp) were used for the simulated assembly dataset. In the case of the GAGE and Assemblathon 1 dataset, N50, N60, N70, N80, and N90 values of a non-reference assembly were used as resolutions.

To assess the relationship between the GMASS score and structural similarity of assembly pairs, the Pearson’s correlation coefficient test was conducted between structural similarities and the GMASS scores calculated using CSBs between simulation datasets. The correlation analyses were also conducted for *L*_*i*_(*A*_1_, *A*_2_), *C*_*i*_(*A*_1_, *A*_2_) and *S*_*i*_ scores in a given resolution *i*. The evolutionary distances which were used for constructing simulated assemblies were treated as indicators of sequence similarity between two assemblies. Information about the evolutionary distance, including phylogenetic tree topology and evolutionary distances between a reference assembly and the simulated assemblies were obtained from [26].

## Results

### Evaluating the GMASS score with simulation data

In this study, it was assumed that if two assemblies are similar, the number and length of CSBs are closer to the number and length of sequence fragments in the assemblies. As shown in Fig. 1b, when two sequences are highly similar, CSBs cover almost the entire sequences, and the total size of CSBs is similar to the one of a smaller sequence (more examples in Additional file 1: Figure S1 and S2). However, CSBs almost covering the entire sequences do not always mean that two assemblies are more similar. If each small region of an assembly is even nearly identical to the counterpart of the other assembly but they are highly rearranged, a large number of small CSBs covering almost the whole of the assemblies can be constructed (Fig. 1c, more examples in Additional file 1: Figure S1 and S2). The GMASS score was developed to provide a single quantitative score measuring the structural similarity of two assemblies by resolving aforementioned issues (Methods).

**Fig. 2:**
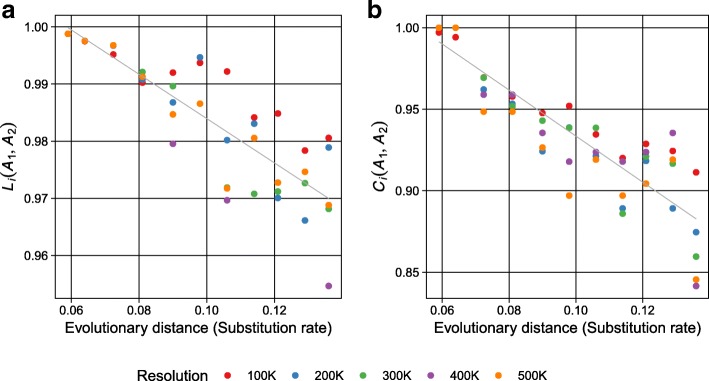
Relationship between evolutionary distance and the similarity of reference against 11 other assemblies obtained from simulation of dataset in terms of the total length of CSBs (*L*_*i*_(*A*_1_, *A*_2_)) (**a**), and the total number of CSBs (*C*_*i*_(*A*_1_, *A*_2_)) (**b**) in several resolutions. The dark gray line represents the linear regression line

The GMASS score was first evaluated using simulated genome assembly dataset (Methods). Starting from an ancestral form of an assembly, descendent assemblies in the dataset were simulated with different evolutionary divergence (substitution rate) which determined the amount of perturbation in the assembly simulation process. Therefore, in this study, the evolutionary divergence was treated as the surrogate of structural difference of two assemblies. As shown in Fig. 2, both *L*_*i*_(*A*_1_, *A*_2_) and *C*_*i*_(*A*_1_, *A*_2_), which represent total length and number of CSBs respectively, showed strong negative correlation with the evolutionary distances in all five resolutions. The Pearson’s correlation coefficients between *L*_*i*_(*A*_1_, *A*_2_) and evolutionary distances were − 0.925 for 100Kbp resolution (*p*-value 4.52E-5), − 0.883 for 200Kbp resolution (*p*-value 3.18E-4), − 0.955 for 300Kbp resolution (*p*-value 4.75E-6), − 0.910 for 400Kbp resolution (*p*-value 9.96E-5), and − 0.949 for 500Kbp resolution (*p*-value 8.67E-6). Similar strong negative correlation was observed in the case of *C*_*i*_(*A*_1_, *A*_2_): − 0.962 for 100Kbp resolution (*p*-value 2.30E-6), − 0.952 for 200Kbp resolution (*p*-value 6.54E-6), − 0.924 for 300Kbp resolution (*p*-value 4.89E-5), − 0.861 for 400Kbp resolution (*p*-value 6.60E-4), and − 0.889 for 500Kbp resolution (*p*-value 2.49E-4). However, as evolutionary distances between assemblies increased, the variance of the scores among different resolutions also increased. Therefore, the decreased pattern of scores were varied by given resolutions.

**Fig. 3:**
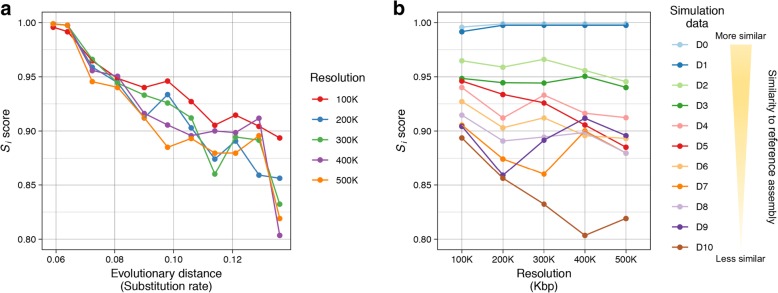
Patterns of the structural similarity score (*S*_*i*_) in terms of evolutionary distance (**a**) and resolution (**b**) using simulated assembly dataset

Next, the *S*_*i*_ score combining *L*_*i*_(*A*_1_, *A*_2_) and *C*_*i*_(*A*_1_, *A*_2_) (Methods) was examined at each different resolution. As shown in Fig. 3a, strong negative correlation was observed from all the five resolutions: Pearson’s correlation coefficient − 0.970 for 100Kbp resolution (*p*-value 7.40E-7), − 0.969 for 200Kbp resolution (*p*-value 9.65E-7), − 0.951 for 300Kbp resolution (*p*-value 7.23E-6), − 0.891 for 400Kbp resolution (*p*-value 2.30E-4), and − 0.924 for 500Kbp resolution (*p*-value 4.71E-5). However, slightly different patterns were observed from different resolutions. To deeply understand the effect of the resolution, the *S*_*i*_ scores calculated from 11 different assemblies were drawn as a function of resolutions (Fig. 3b). More obvious variations of scores among different resolutions were found especially in the case where the evolutionary distance became larger.

**Fig. 4:**
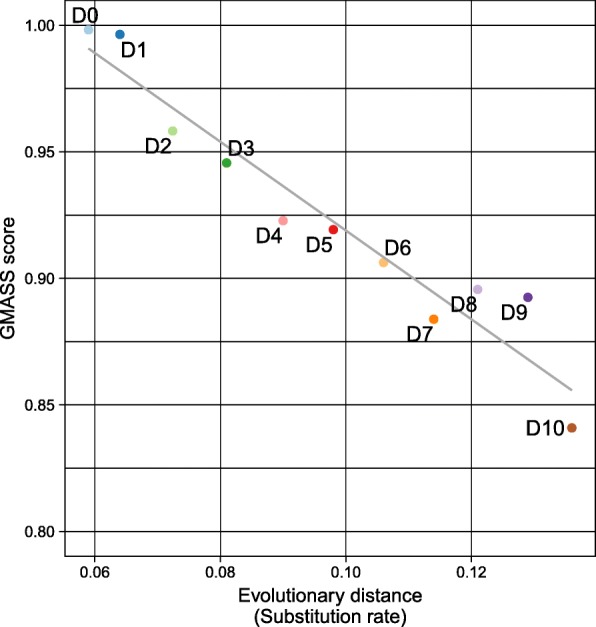
Relationship between evolutionary distance and the GMASS score using simulated assembly dataset. The dark gray line represents the linear regression line

Finally, the GMASS scores were calculated by averaging the *S*_*i*_ scores from all five resolutions, and comparison made in terms of evolutionary divergences (Fig. 4). Strong negative correlation between the GMASS score and evolutionary distance was observed (Pearson’s correlation coefficient − 0.962, *p*-value 2.15E-6). There was one exception of the score of D7 which was lower than the ones of D8 and D9, despite being more divergent than D7. This was because larger number of CSBs were constructed from D7 than D8 and D9, although the coverages of CSBs were similar in all three assemblies (Additional file 2: Table S1).

### Application of the GMASS score to assembly benchmark data

The GMASS score was used to compare each pair of assemblies constructed in the GAGE [18] and Assemblathon 1 [20] dataset (Methods). The statistics of the assemblies were quite varied even though they were constructed using the same sequencing datasets (Additional file 2: Table S2).

In the case of the GAGE dataset, the GMASS score between the same assembly was approximately 1, and the average of the GMASS scores between different assemblies was 0.537 with standard deviation 0.188 (Table 1). The resolution specific score *S*_*i*_ of resolution *i* calculated from two different assemblies was also varying (Additional file 1: Figure S3, Additional file 2: Table S3). For example, the GMASS score was 0.087 between the MSR-CA assembly and Velvet assembly. The maximum *S*_*i*_ score between them was 0.4126 when N90 of a non-reference assembly was used as a resolution. However, the minimum score was only 0.0003 when N50 of a non-reference assembly was used as a resolution (see Methods for the selection of resolutions). In the case of the N90 resolution, 253 CSBs were constructed, and their coverages of compared scaffolds greater than the resolutions of the MSR-CA assembly and Velvet assembly were 0.5023 and 0.4188, respectively (Additional file 2: Table S4). When syntenic relationship was analyzed using the Circos plots drawn by mySyntenyPortal [36], several short CSBs were constructed between one scaffold of one assembly, especially large one, and multiple scaffolds of the other assembly (Additional file 1: Figure S4). However, in the case of the N50 resolution, although 29 scaffolds of the MSR-CA assembly and 51 scaffolds of the Velvet assembly were used to make CSBs, only one CSB was constructed between them. Its coverage of scaffolds used for comparison was 0.0249 for the MSR-CA assembly and 0.0037 for the Velvet assembly.

**Table 1:** The GMASS scores of assembly pairs obtained from the GAGE dataset

ABySS2	0.992							
ALLPATHS-LG	0.671	1.000						
Bambus2	0.660	0.523	1.000					
CABOG	0.689	0.620	0.446	1.000				
MSR-CA	0.662	0.447	0.376	0.419	1.000			
SGA	0.687	0.706	0.740	0.759	0.701	0.984		
SOAPdenovo	0.665	0.731	0.542	0.595	0.589	0.734	0.993	
Velvet	0.457	0.134	0.250	0.259	0.087	0.499	0.384	1.000
	ABySS2	ALLPATHS-LG	Bambus2	CABOG	MSR-CA	SGA	SOAPdenovo	Velvet

In the case of Assemblathon 1 dataset, the average GMASS score was lower than the score from the GAGE dataset (Table 2, average = 0.47, standard deviation = 0.158). The minimum GMASS score of 0.274 was obtained between assemblies from WTSI-S and CSHL. The highest GMASS score was only 0.647 obtained between assemblies from WTSI-S and Broad. The features of constructed CSBs, *C*(*A*_1_, *A*_2_), *L*(*A*_1_, *A*_2_) and *S*_*i*_ scores were also different when difference resolutions were used (Additional file 1: Figure S5, Additional file 2: Table S5 and S6). The GMASS scores calculated using the self-alignments of assemblies showed almost 1 in both cases of GAGE and Assemblathon 1.

**Table 2:** The GMASS scores of assembly pairs obtained from the Assemblathon 1 dataset. The teams’ names constructed each assembly are written in parentheses

ALLPATHS-LG (Broad)	1.000				
ABySS (BGI)	0.642	1.000			
SOAPdenovo (DOEJGI)	0.370	0.632	1.000		
Meraculous (WTSI-S)	0.647	0.607	0.557	1.000	
Celera (CSHL)	0.333	0.313	0.327	0.274	1.000
	ALLPATHS-LG (Broad)	ABySS (BGI)	SOAPdenovo (DOEJGI)	Meraculous (WTSI-S)	Celera (CSHL)

## Discussion

The GMASS score was designed to represent the degree of structural similarity between a pair of assemblies. This new measure was developed based on the distribution pattern of CSBs between a pair of assemblies, which were identified by applying the concept of synteny block in comparative genomics. With pairwise alignment results, CSBs are constructed with algorithms for constructing synteny blocks, the GMASS scores are then calculated by considering both the length and number of CSBs.

Based on evaluation with simulated dataset, we found that the GMASS score was highly related to the structural similarity. However, the GMASS score of the D7 assembly in the dataset was lower than the scores of the D8 and D9 assembly, which were more divergent than D7 from a reference assembly. In this case, larger numbers of CSBs were constructed from the D7 assembly than the D8 and D9 assembly, but the CSB coverage of D7 was higher than D8 and D9. It was as a result of the high rearrangement of small regions in the D7 assembly which are nearly identical to the counterpart of the reference assembly. This kind of rearrangements in small regions were mostly observed in D7 than D8 and D9 (Additional file 1: Figure S6). The CSBs pattern in other resolutions can be identified from our supplementary website (http://bioinfo.konkuk.ac.kr/GMASS/).

From the GMASS scores calculated using the assembly benchmark datasets (GAGE and Assemblathon 1), we found that the current assembly tools generated different assemblies even though the same sequencing read datasets are used. For example, the GMASS score between Velvet and ALLPATHS-LG assembly in the GAGE dataset, which were both developed based on the *de Bruijn* graph, was very low. This may indicate that the quality of assembly is not only affected by the graph and traversal algorithm, but also by additional pre- and post-processing algorithms and parameters used in the assembler. In the case of the GAGE dataset, the GMASS scores calculated using the self-alignments of the ABySS2, SGA and SOAPdenovo assembly were not 1 (Table 1). The reason is that there were short scaffolds with high fraction of repetitive sequences, which were masked out in a repeat masking step, and eventually ignored during sequence alignment for the CSB construction.

The GMASS scores can be used in structural-level similarity analysis between diverse assemblies as well as genomes of multiple species or individuals. Especially, because the score was significantly related to the evolutionary distance, it can be also used as alternative evolutionary distances among species or individuals by complementing traditional nucleotide-level similarity measures, such as ANI [37] and GBDP [38]. This measure can also be used to infer the accuracy of a novel assembly by calculating the score between the assembly and true assembly for target species. In addition, the relative improvement of a new assembler against existing assemblers can be approximately inferred by comparing the GMASS scores which are calculated from true assembly against assemblies created by the new assembler and existing assemblers.

The CSBs of different assemblers can be used to improve or develop the assemblers. For example, researchers can identify regions with structural differences, which are not covered by CSBs among assemblies built by different assemblers. These regions might be too complicated to assemble using existing assemblers or assembly algorithms, therefore new assemblers need to be developed to assembly such regions more accurately.

Resolution is a critical factor to consider when calculating the GMASS score. The resolution makes it difficult to compare scaffolds shorter than a given resolution by ignoring them in the construction of CSBs. In addition, the contiguity of an assembly was quite variable according to the result displayed by GAGE and Assemblathon datasets, it is not possible to use a fixed set of resolutions. Therefore, we used a dynamic set of resolutions for different assembly pairs based on the statistics of scaffold lengths (N50, N60, N70, N80, and N90) of a non-reference assembly for the GAGE and Assemblathon 1 datasets. However, a large fraction of sequences is ignored if a large value is used as a resolution. Further studies to define appropriate resolutions are required.

## Conclusions

The GMASS score is a novel measure for representing structural similarity between two assemblies. The usability of the GMASS score was successfully evaluated using various assembly datasets. The novel measure will contribute to the understanding of assembly output and developing de novo assemblers.

## Additional files

Additional file 1: **Figure S1.** Examples of different patterns of CSBs constructed among human (GRCh38/hg38 assembly) and chimpanzee (Pan_tro 3.0/panTro5 assembly) chromosomes. Linear plots were drawn by mySyntenyPortal (https://github.com/jkimlab/mySyntenyPortal/). **Figure S2.** Examples of different patterns of CSBs constructed among human (GRCh38/hg38 assembly) and mouse (GRCm38/mm10 assembly) chromosomes. Linear plots were drawn by mySyntenyPortal (https://github.com/jkimlab/mySyntenyPortal/). **Figure S3.** Patterns of *L*_*i*_(*A*_1_, *A*_2_), *C*_*i*_(*A*_1_, *A*_2_) and *S*_*i*_ scores of assembly pairs obtained from the GAGE dataset in different resolutions. **Figure S4.** Circos plots representing CSBs constructed between the MSR-CA and Velvet assembly in five different resolutions. N50 (843,765 bp), N60 (669,249 bp), N70 (484,156 bp), N80 (298,277 bp), and N90 values (89,649 bp) of a more fragmented Velvet assembly are used as the resolution. Colored and white boxes represent the scaffolds of the MSR-CA and Velvet assembly respectively. **Figure S5.** Patterns of *L*_*i*_(*A*_1_, *A*_2_), *C*_*i*_(*A*_1_, *A*_2_) and *S*_*i*_ scores of assembly pairs obtained from the Assemblathon 1 dataset in different resolutions. **Figure S6.** Linear plots representing CSBs between a reference assembly (R_seq) and simulated assemblies (D7_seq, D8_seq and D9_seq) in 300 K resolution. (DOCX 4759 kb)

Additional file 2: **Table S1.** Statistics of a reference assembly and 11 descendant assemblies, as well as CSBs constructed between them. AS denotes the scaffolds contained each assembly, and SF represents the CSBs constructed between each assembly and the reference assembly. **Table S2.** Statistics of analyzed assemblies and reference sequences. In the case of the GAGE dataset, the statistics of the reference sequences were calculated using the human chromosome 14 (HG38/GRCh38 assembly) downloaded from the UCSC genome browser [30]. In the case of the Assemblathon 1 dataset, the teams’ names constructed each assembly are written in parentheses, and the statistics of reference are the averages of statistics of two haplotypes used in the study. **Table S3.** *L*_*i*_(*A*_1_, *A*_2_), *C*_*i*_(*A*_1_, *A*_2_) and *S*_*i*_ scores of assembly pairs obtained from the GAGE dataset in different resolutions. In the “Combination” column, the name of an assembly used as reference appears first. **Table S4.** Statistics of each assembly and CSBs of assembly pairs obtained from the GAGE dataset. AS denotes the scaffolds in each assembly, and SF represents the CSBs constructed between each assembly pair. In combination column, front assembly is used as reference assembly and rear assembly is used as target assembly. **Table S5.** *L*_*i*_(*A*_1_, *A*_2_), *C*_*i*_(*A*_1_, *A*_2_) and *S*_*i*_ scores of assembly pairs obtained from the Assemblathon 1 dataset in different resolutions. In the “Combination” column, the name of an assembly used as reference appears first. **Table S6.** Statistics of each assembly and CSBs of assembly pairs obtained from the Assemblathon1 dataset. AS denotes the scaffolds in each assembly, and SF represents the CSBs constructed between each assembly pair. In combination column, front assembly is used as reference assembly and rear assembly is used as target assembly. Used assemblers are omitted. (XLSX 84 kb)

## Abbreviations

CSBs: Consensus segment blocks
NGS: Next-generation sequencing
