# Supplementary material for: GMASS: a novel measure for genome assembly structural similarity
Source: BMC Bioinformatics. 2019 Mar 18;20:147. doi: 10.1186/s12859-019-2710-z (PMC6423833; doi:10.1186/s12859-019-2710-z)
Supplement: Supplementary file 1 — Figure S1. Examples of different patterns of CSBs constructed among human (GRCh38/hg38 assembly) and chimpanzee (Pan_tro 3.0/panTro5 assembly) chromosomes. Linear plots were drawn by mySyntenyPortal (https://github.com/jkimlab/mySyntenyPortal/). Figure S2. Examples of different patterns of CSBs constructed among human (GRCh38/hg38 assembly) and mouse (GRCm38/mm10 assembly) chromosomes. Linear plots were drawn by mySyntenyPortal (https://github.com/jkimlab/mySyntenyPortal/). Figure S3. Patterns of Li(A1, A2), Ci(A1, A2) and Si scores of assembly pairs obtained from the GAGE dataset in different resolutions. Figure S4. Circos plots representing CSBs constructed between the MSR-CA and Velvet assembly in five different resolutions. N50 (843,765 bp), N60 (669,249 bp), N70 (484,156 bp), N80 (298,277 bp), and N90 values (89,649 bp) of a more fragmented Velvet assembly are used as the resolution. Colored and white boxes represent the scaffolds of the MSR-CA and Velvet assembly respectively. Figure S5. Patterns of Li(A1, A2), Ci(A1, A2) and Si scores of assembly pairs obtained from the Assemblathon 1 dataset in different resolutions. Figure S6. Linear plots representing CSBs between a reference assembly (R_seq) and simulated assemblies (D7_seq, D8_seq and D9_seq) in 300 K resolution. (DOCX 4759 kb) [file 12859_2019_2710_MOESM1_ESM.docx]

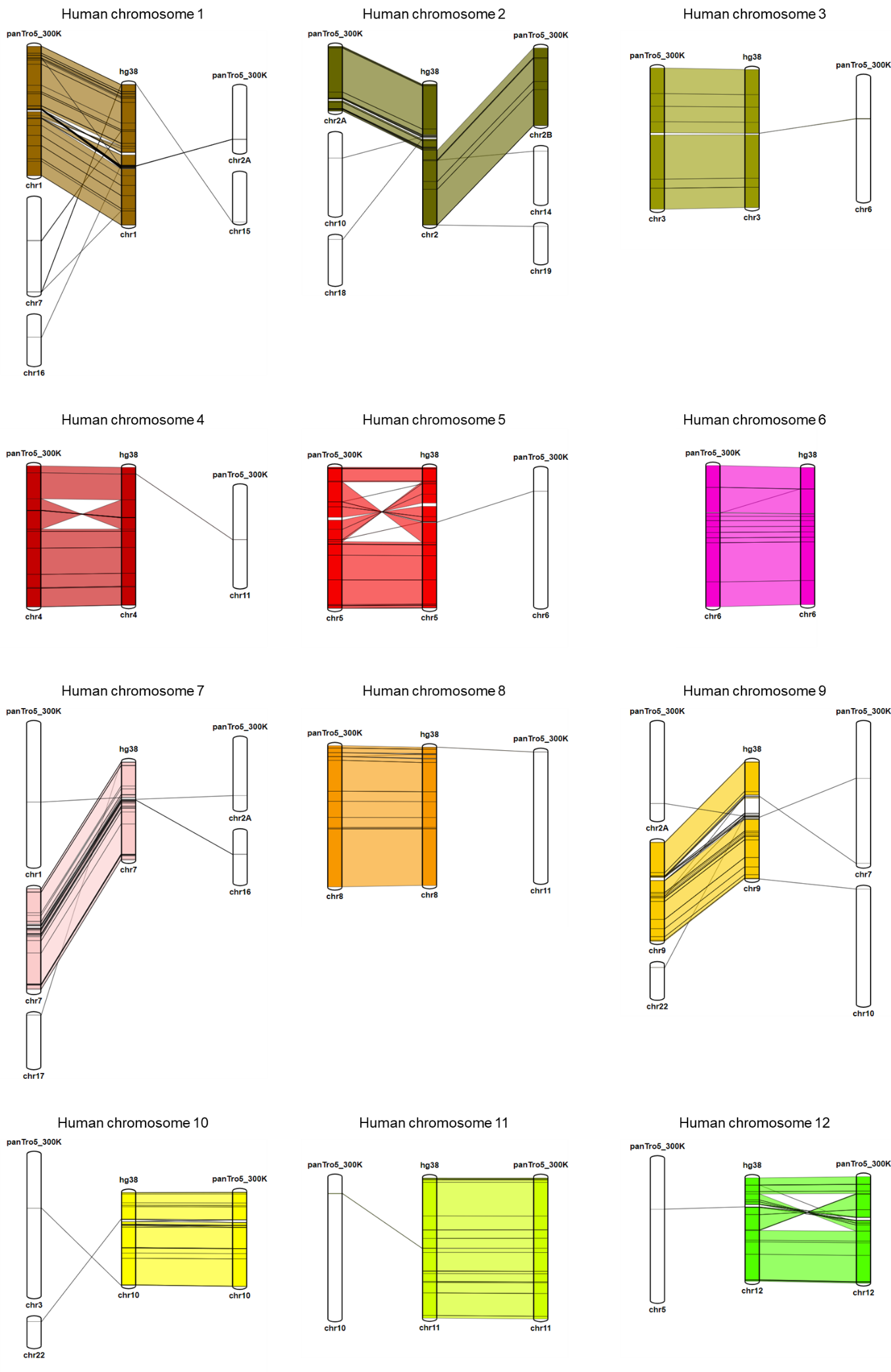


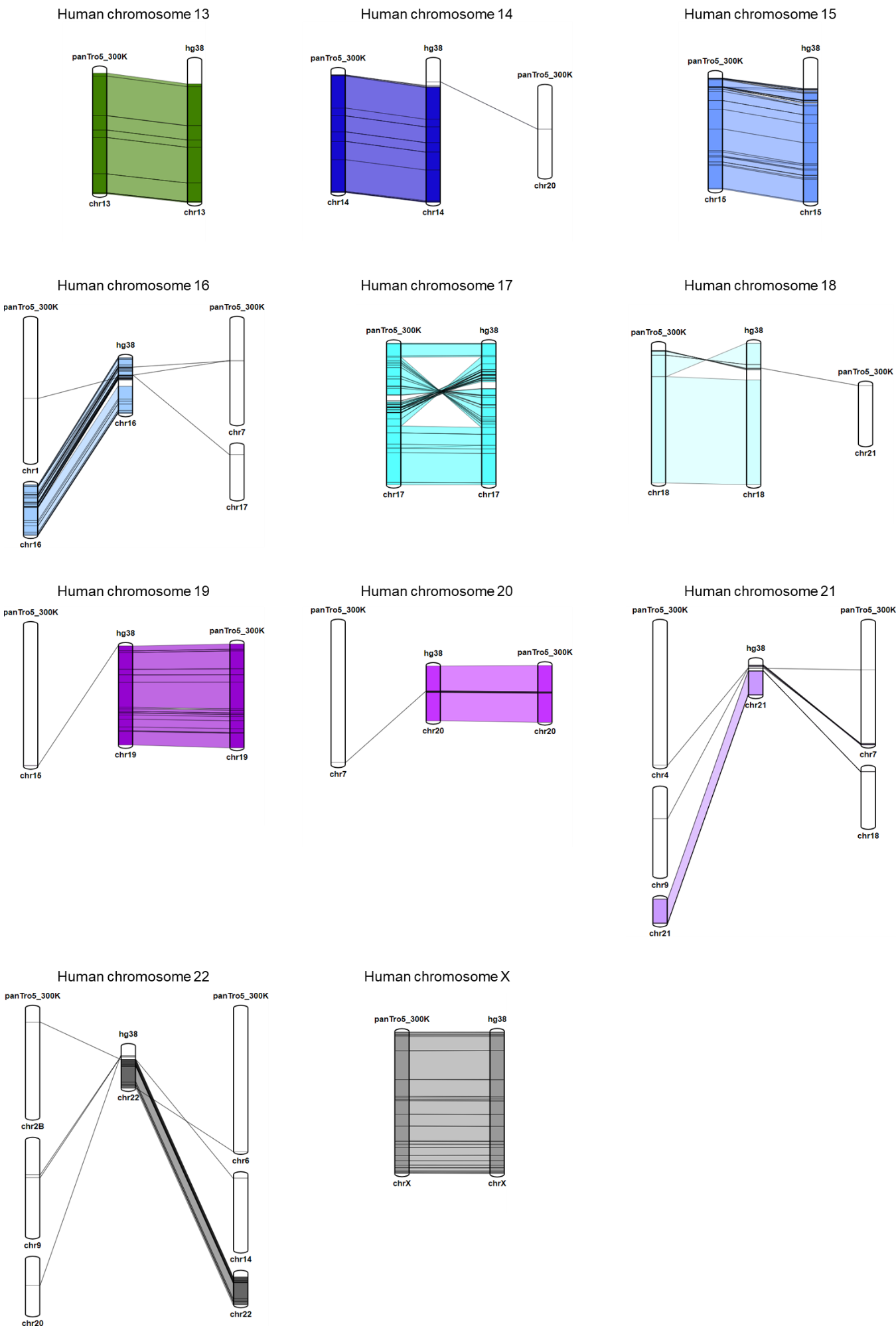


**Fig. S1** Examples of different patterns of CSBs constructed among human (GRCh38/hg38 assembly) and chimpanzee (Pan_tro 3.0/panTro5 assembly) chromosomes. Linear plots were drawn by mySyntenyPortal (https://github.com/jkimlab/mySyntenyPortal/).


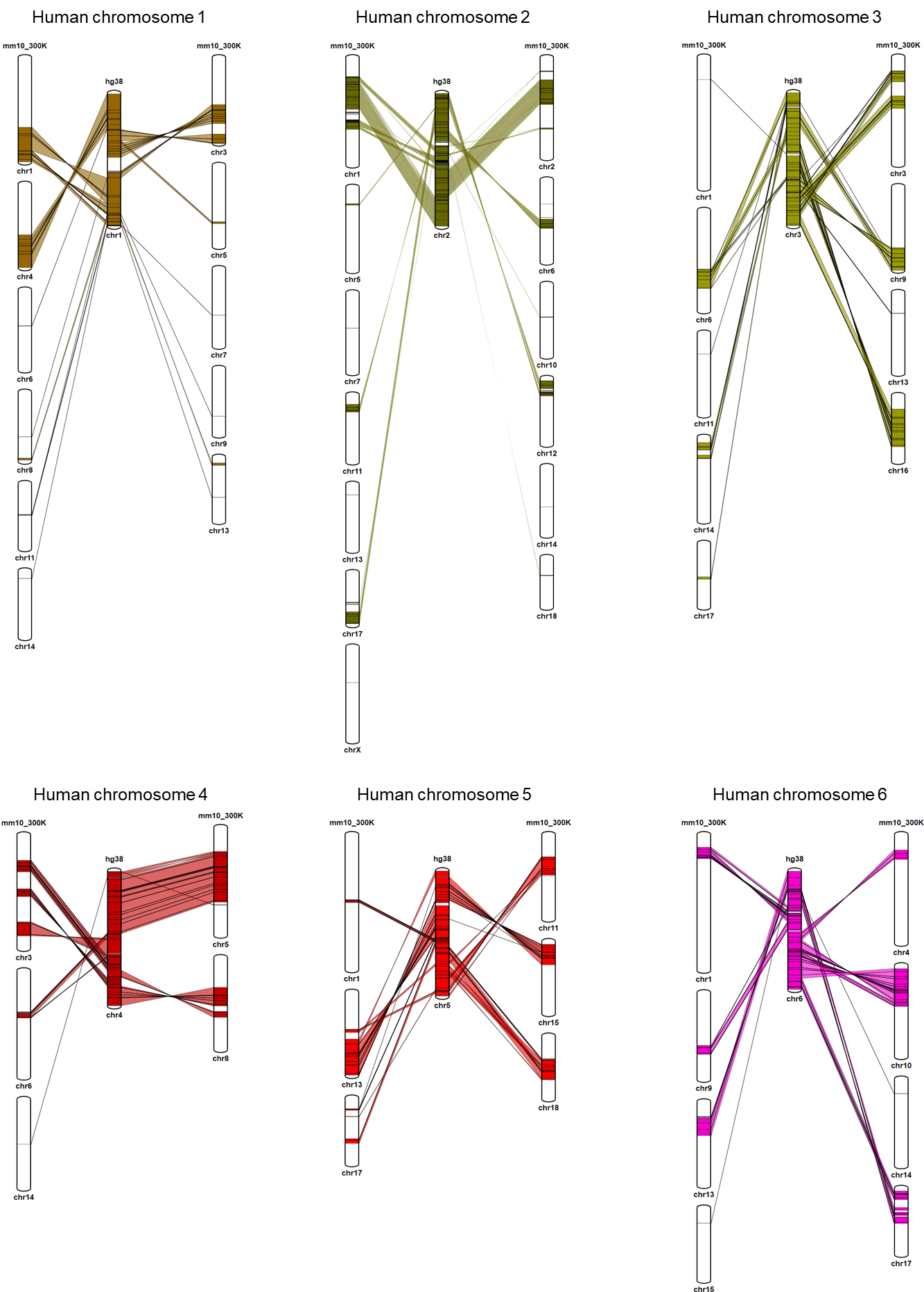


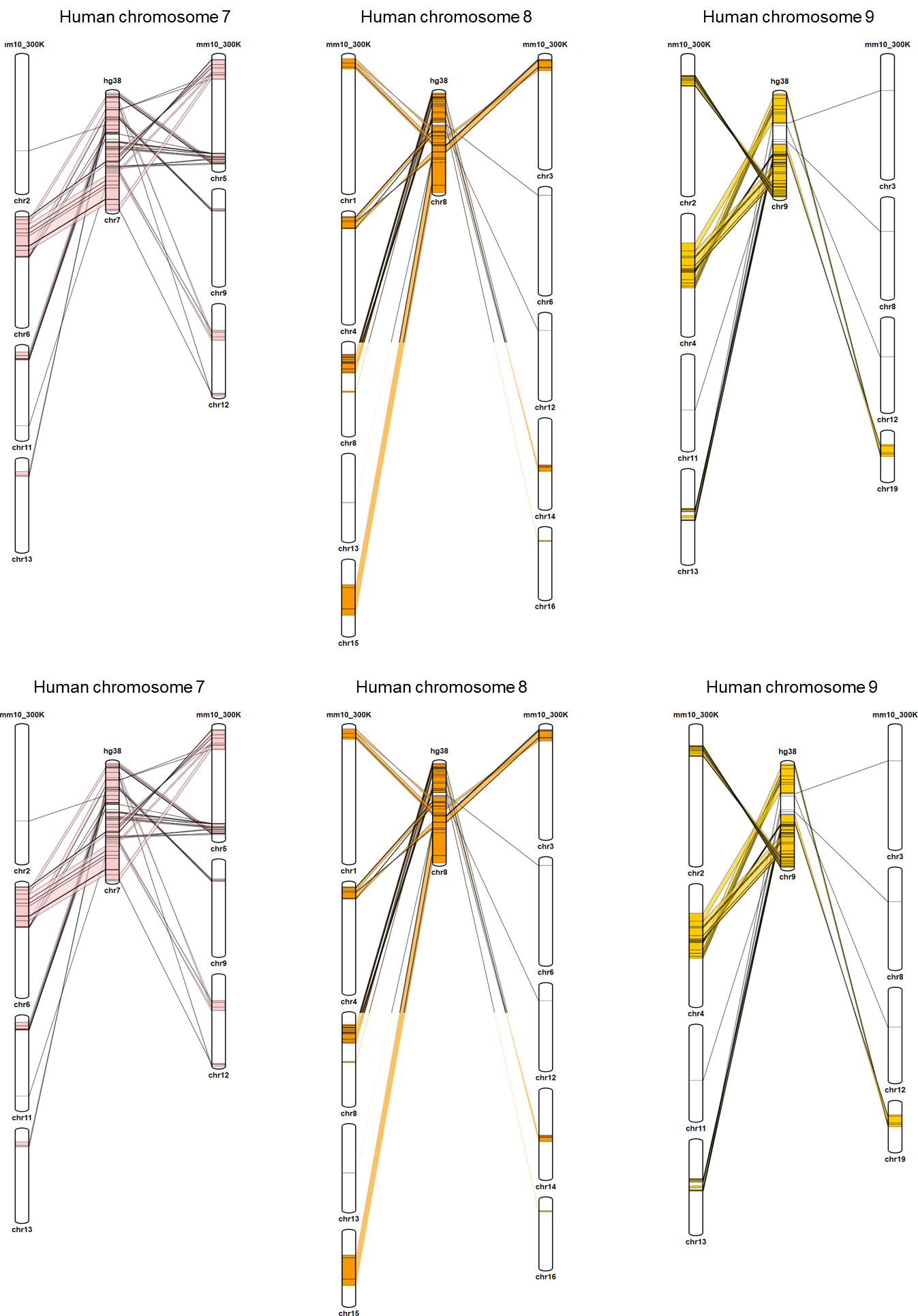

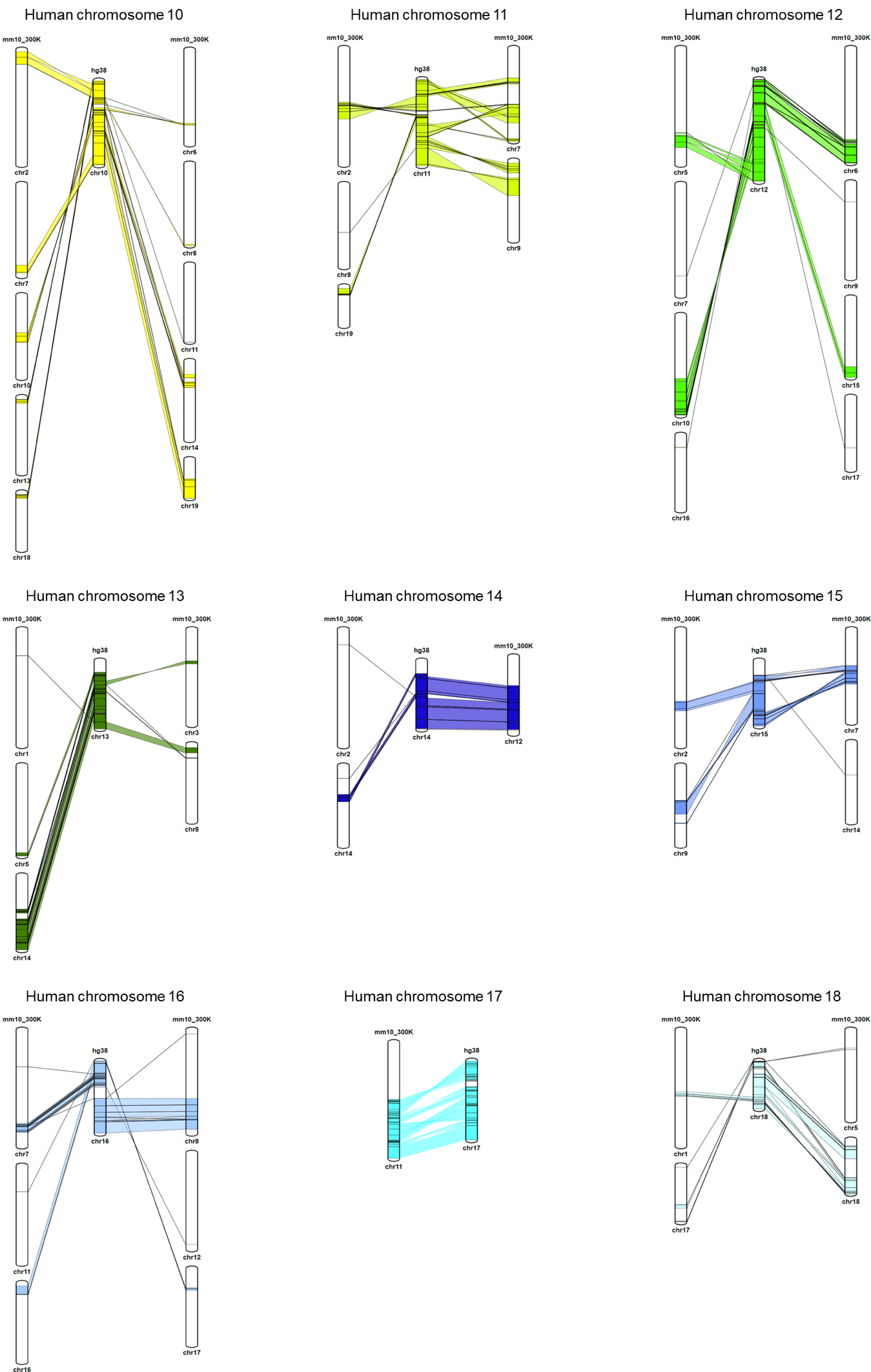


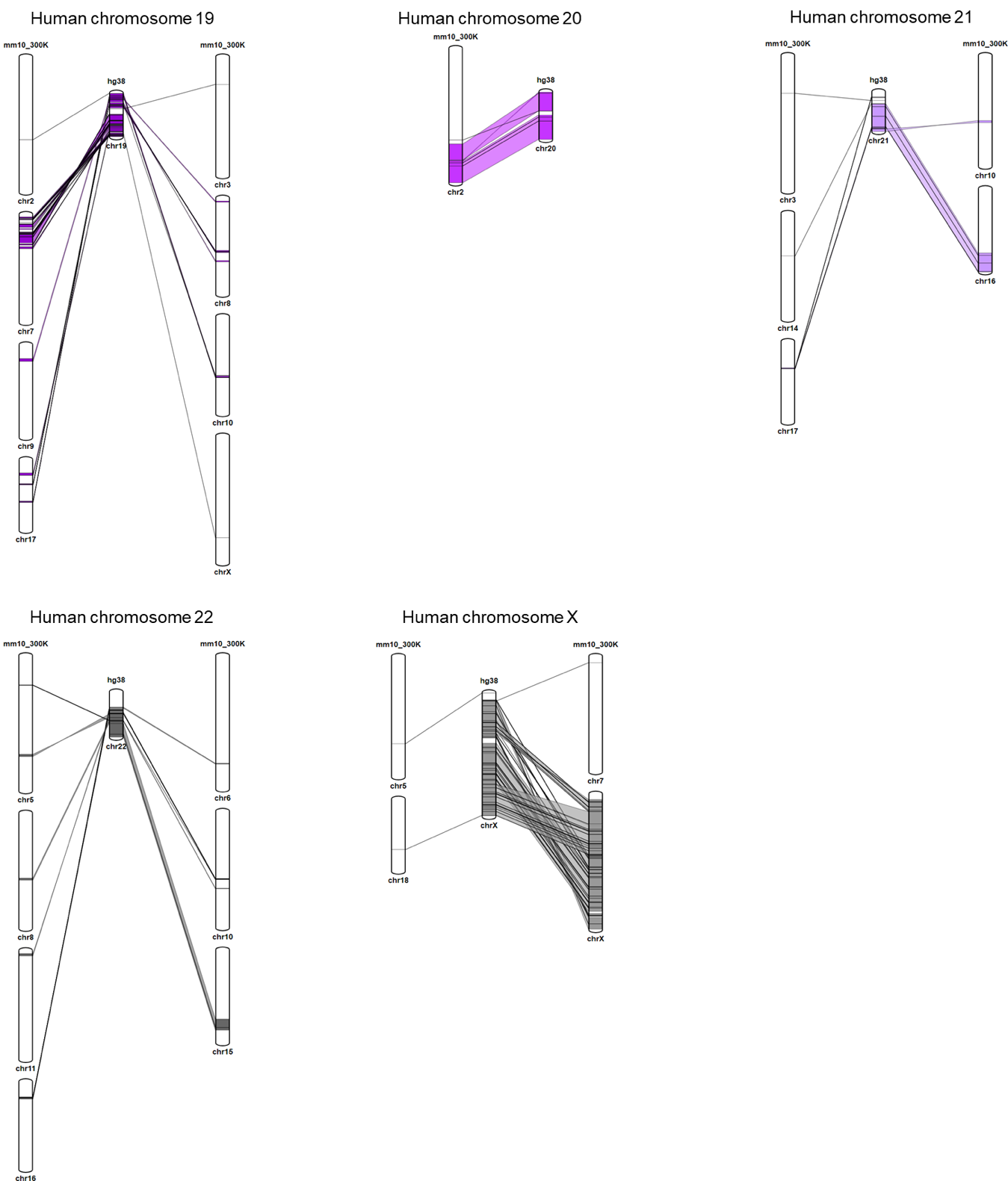


**Fig. S2** Examples of different patterns of CSBs constructed among human (GRCh38/hg38 assembly) and mouse (GRCm38/mm10 assembly) chromosomes**.** Linear plots were drawn by mySyntenyPortal (https://github.com/jkimlab/mySyntenyPortal/).

a

b

c

**Fig. S3** Patterns of $L_{i}\left( A_{1},A_{2} \right)$, $C_{i}\left( A_{1},A_{2} \right)$ and $S_{i}$ scores of assembly pairs obtained from the GAGE dataset in different resolutions.


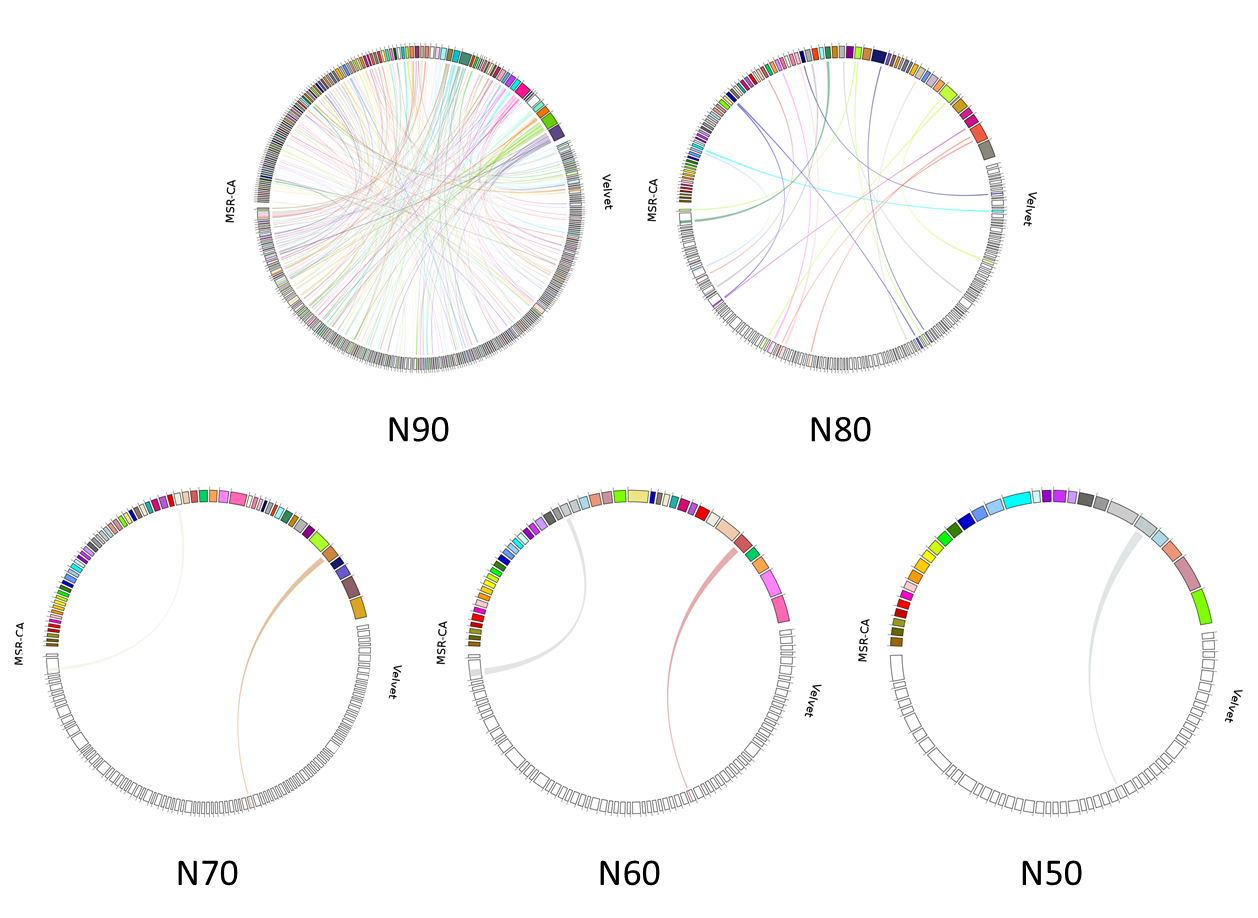


**Fig. S4** Circos plots representing CSBs constructed between the MSR-CA and Velvet assembly in five different resolutions. N50 (843,765bp), N60 (669,249bp), N70 (484,156bp), N80 (298,277bp), and N90 values (89,649bp) of a more fragmented Velvet assembly are used as the resolution. Colored and white boxes represent the scaffolds of the MSR-CA and Velvet assembly respectively.

a

b

c

**z**

**Fig. S5** Patterns of $L_{i}\left( A_{1},A_{2} \right)$, $C_{i}\left( A_{1},A_{2} \right)$ and $S_{i}$ scores of assembly pairs obtained from the Assemblathon 1 dataset in different resolutions.


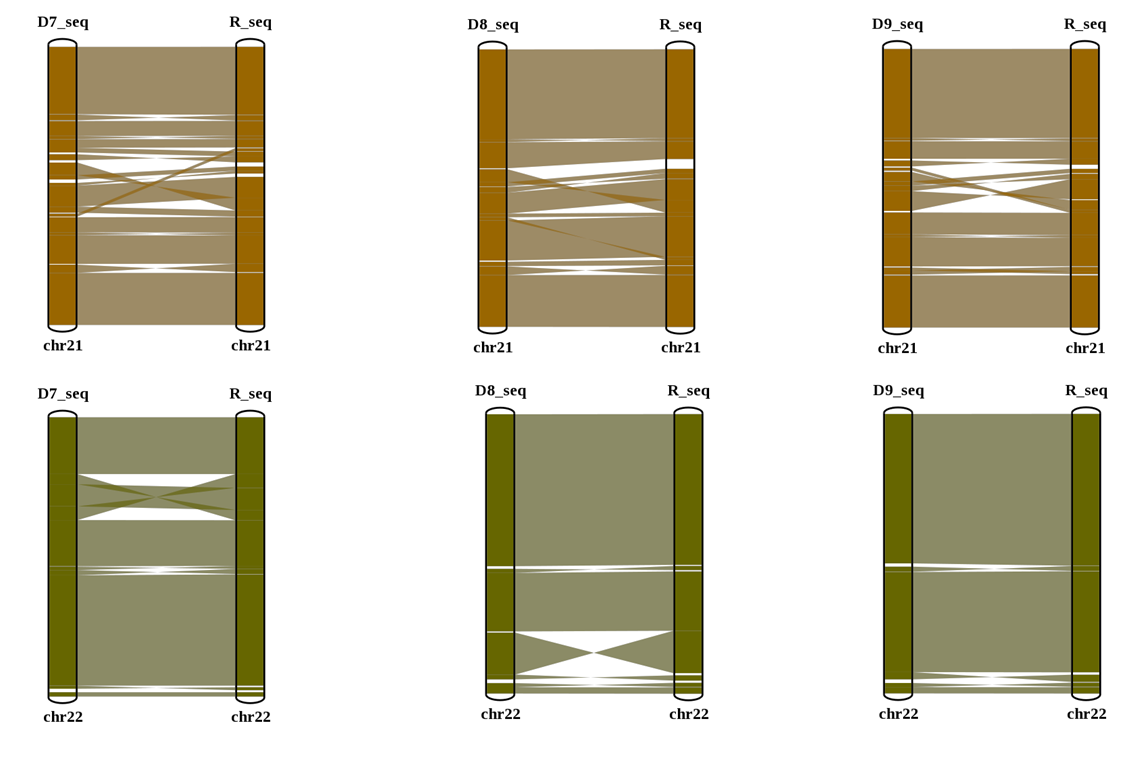


**Fig. S6** Linear plots representing CSBs between a reference assembly (R_seq) and simulated assemblies (D7_seq, D8_seq and D9_seq) in 300K resolution.
